# Cochlear-implant listeners benefit from training with time-compressed speech, even at advanced ages

**DOI:** 10.1121/10.0025431

**Published:** 2024-05-02

**Authors:** Amara C. Ezenwa, Matthew J. Goupell, Sandra Gordon-Salant

**Affiliations:** Department of Hearing and Speech Sciences, University of Maryland, College Park, Maryland 20742, USA acezenwa@umd.edu, goupell@umd.edu, sgsalant@umd.edu

## Abstract

This study evaluated whether adaptive training with time-compressed speech produces an age-dependent improvement in speech recognition in 14 adult cochlear-implant users. The protocol consisted of a pretest, 5 h of training, and a posttest using time-compressed speech and an adaptive procedure. There were significant improvements in time-compressed speech recognition at the posttest session following training (>5% in the average time-compressed speech recognition threshold) but no effects of age. These results are promising for the use of adaptive training in aural rehabilitation strategies for cochlear-implant users across the adult lifespan and possibly using speech signals, such as time-compressed speech, to train temporal processing.

## 1. Introduction

Age-related temporal-processing deficits in older adult listeners affect their ability to perform a myriad of auditory processing tasks relative to that of younger adult listeners. For example, older adults generally experience difficulty in speech recognition, which is exacerbated with time-compressed or rapid speech (Gordon-Salant and Fitzgibbons, 2001). Even in older normal-hearing adults, there are age-related changes in peripheral processing, central auditory processing, and cognitive abilities that are associated with reduced speech recognition in difficult listening situations (Füllgrabe *et al.*, 2015). Aging of the peripheral auditory system and long durations of limited auditory stimulation lead to spiral ganglion cell loss (Kujawa and Liberman, 2015). In the central auditory system, hearing impairment may contribute to loss and limited activation of neurons, for example, in the inferior colliculus and auditory cortex (Middlebrooks and Snyder, 2010; Kirby and Middlebrooks, 2012). Central deficits impact temporal representations of auditory signals, resulting in a less precise, degraded signal (Anderson *et al.*, 2012; Anderson *et al.*, 2022).

Cochlear implants (CIs) partially restore hearing and improve speech recognition in listeners with up to profound hearing losses, even among adults of advanced ages (Lenarz *et al.*, 2012). Like older acoustic-hearing listeners, older CI listeners may experience greatly diminished speech recognition in challenging situations such as in the presence of background noise, competing speech, reverberation, and with time-compressed speech (e.g., Ji *et al.*, 2013). Furthermore, the spectral degradation that occurs with a CI exacerbates the age-related deficits in time-compressed speech recognition (Tinnemore *et al.*, 2022).

CI users also experience a period of improving speech recognition over months to years after activation, which is thought to be a result of audiologists optimizing the devices' programming, and the users' learning and plasticity (Blamey *et al.*, 2013); this improvement can be facilitated with aural rehabilitation and training (Fu and Galvin, 2008). As the improvement in speech recognition after activation appears to occur at a slower rate for older compared to younger CI users (Canfarotta *et al.*, 2020), it could be that training is even more important for older CI users who experience age-related temporal-processing deficits. The brain retains some plasticity, and older listeners retain some ability to demonstrate perceptual learning (Bieber and Gordon-Salant, 2021). For example, auditory training improved temporal rate discrimination in two older (≥65 years of age) CI listeners of the six tested when presented with single-electrode stimulation (Goldsworthy and Shannon, 2014). Rate discrimination also improved for acoustic-hearing listeners when presented band-limited pulse trains; furthermore, older acoustic-hearing listeners improved temporal-processing abilities to levels of untrained younger listeners (Anderson *et al.*, 2022). However, the benefit of auditory training focused on improving the processing of speech signals that stress rapid temporal processing has yet to be assessed in older CI listeners. As noted above, one form of challenging speech for older CI listeners is time-compressed speech (Ji *et al.*, 2013; Tinnemore *et al.*, 2022), which may be appropriate as a training stimulus to improve auditory temporal processing in older adults (Manheim *et al.*, 2018).

The current study is an initial effort to evaluate the benefit of an adaptive training paradigm with time-compressed speech for adult CI listeners across the lifespan. We hypothesized that listeners would demonstrate improved recognition of rapid speech after completion of auditory training, based on previous studies showing improved pulse-rate discrimination following training (Goldsworthy and Shannon, 2014; Bissmeyer *et al.*, 2020; Anderson *et al.*, 2022). Although older CI listeners were expected to demonstrate improved recognition of rapid speech following training, it was also hypothesized that the training gains would diminish with increasing age (i.e., there would be an age by training interaction resulting in more modest training gains for older listeners). Finally, we hypothesized that improvement in recognition of rapid speech would generalize to better speech recognition in noise post-training, given the assumption that training with rapid speech improves auditory temporal processing, which also appears to underlie speech recognition in noise (Frisina and Frisina, 1997).

## 2. Method

### 2.1 Listeners

This study recruited 14 CI listeners, ranging in age from 30 to 82 years old (mean = 57.4 years old), who wore CIs for at least 1 year. All listeners were native English speakers and postlingually deafened. They had minimal residual acoustic hearing and self-reported no usable acoustic hearing. Some were verified to have no responses to audiometric equipment limits or thresholds >80 dB hearing level (HL; ANSI, 2018) bilaterally as measured by a research audiologist in the laboratory or by a clinical audiologist. The listeners were unilaterally or bilaterally implanted with Cochlear Ltd. (Sydney, Australia) devices, except for S7 who used a MED-EL (Innsbruck, Austria) device. The listeners were required to meet the following additional criteria for enrollment into the study: a minimum word recognition score of 20% while wearing their device(s), a passing score of at least 22 on the Montreal Cognitive Assessment (MoCA; Nasreddine *et al.*, 2005), and a high school level of education. Table 1 presents demographic information [sex, age, score on the MoCA, duration of deafness (defined as the time between the estimated onset of severe-to-profound hearing loss to CI activation), duration of CI use, and consonant-nucleus-consonant (CNC) word score while using the CI] for the individual CI listeners. The research reported in this article was approved by the Institutional Review Board for Human Subjects at the University of Maryland. Informed consent was obtained from the listeners.

**Table 1.** Demographic information for the listeners.

Code	Sex	Age (yr)	MoCA	Duration of deafness (yr)		Duration of CI use (yr)		CNC word score (%)
				Left	Right	Left	Right	
S1	M	30	28		1		12	82
S2	F	32	28	2	20	30	12	94
S3	M	35	25	12	8	13	17	90
S4	M	49	24		1		21	36
S5	F	54	30	1	0	14	15	92
S6	F	58	30	1	5	14	10	92
S7	F	60	30		33		10	76
S8	F	63	29	10	11	13	12	68
S9	F	64	28	5	0	11	16	90
S10	F	69	28	12	13	12	11	90
S11	F	71	28	2	1	16	17	99
S12	F	76	29	0	4	22	17	64
S13	M	82	28	1	50	19	11	60
S14	M	82	27	3	7	19	13	96

### 2.2 Stimuli

Test stimuli were sentences from the Institute of Electrical and Electronics Engineers (IEEE) corpus (Rothauser *et al.*, 1969; e.g., “Rice is often served in round bowls.”) and AzBio sentences (Spahr *et al.*, 2012; e.g., “His organizational skills were lacking.”). IEEE sentences are phonetically balanced sentences with generally low word-context predictability. The recordings were made with two male talkers, a novel talker whose recordings were used only at the pretest and posttest sessions and a training talker whose recordings were used in all pretest, training, and posttest sessions. The 720 IEEE sentences were time compressed at rates from 5% to 95% in 5% increments using Praat software (Boersma and Weenink, 2013). The spectral distribution of the original speech signals was retained following time compression. AzBio sentences are phonemically balanced sentences spoken by two female and two male native English talkers. Each list consists of 20 fixed-intensity sentences that were presented in 10-talker babble noise. These sentences are often used clinically, specifically for determining CI candidacy and monitoring patient progress with the CI postimplantation. For this study, equally intelligible sentence lists 3, 5, 8, and 9 (Schafer *et al.*, 2012) were randomly selected for the listener at the pretest and posttest sessions.

### 2.3 Procedures

All pretest, training, and posttest measures took place in a sound-treated booth or quiet room. The stimuli were presented via circumaural headphones (HD650, Sennheiser, Hannover, Germany) worn over the listener's sound processors with sentence materials presented bilaterally. Stimuli were calibrated to a volume of 60 dB-A and adjusted according to the level indicated by the listener to be audible and most comfortable. Additionally, the settings and programs of the listener's sound processor(s) were recorded at the pretest session. Volume settings obtained at the pretest session were maintained for the duration of the study. Earplugs were worn in each ear canal to prevent changes in acoustics due to resonances in the ear canal and avoid the contribution of residual hearing (although this was a minimal concern for this study given that the listeners had negligible acoustic hearing).

AzBio sentences were presented with multi-talker babble noise at a fixed signal-to-noise ratio (SNR) of +10 dB with no time compression. The listener's task was to repeat the sentences that they heard. Percent correct scores were based on how many keywords in the sentence list that the listener repeated correctly and calculated as a percentage of total words correct divided by total words presented.

IEEE sentences were presented in quiet using an adaptive procedure to determine the time-compression threshold corresponding to 50% correct performance. The five key words in each IEEE sentence were scored individually as correct or incorrect. The adaptive procedure increased the time-compression ratio (faster sentence) following three or more words repeated correctly and decreased the time-compression ratio (slower sentence) following three or more words repeated incorrectly. The first sentence was presented at a 20% time-compression ratio with an initial step size of 10%. After the second reversal, the step size was reduced to 5%. There were 30 randomly selected sentences presented in each block to determine the time-compression threshold. Each listener's time-compression threshold at 50% performance was derived using a psychometric fitting function implemented in MATLAB (version 2020a, the Mathworks, Natick, MA).

#### 2.3.1 Pretest session

During the pretest visit, listeners completed an audiological assessment, a case history, and a cognitive screening to determine if they met the criteria for the study. Once enrolled, they participated in speech recognition testing during the pretest. First, they listened to two AzBio sentence lists with 20 sentences per list, presented in multi-talker babble noise at a +10 dB SNR. The listener's task was to repeat the sentence that they heard. The second test consisted of presentation of two blocks of the IEEE sentences in quiet with 30 sentences per block. The first block used the sentences recorded by the novel talker, and the second block used the sentences recorded by the training talker. The listener's task was to repeat each sentence that they heard, and the adaptive procedure described above was used. Listeners did not receive correct-answer feedback during the pretest session.

#### 2.3.2 Training sessions

Listeners returned to the labortory for a total of five training sessions that occurred over the course of 1–2 weeks (3–5  training sessions per week). During each training session, the listener was presented with 3 blocks of IEEE sentences, 30 sentences per block, with the training talker used for all 3 blocks. The listener repeated each sentence, which was followed by correct-answer feedback. During feedback, the listener heard the sentence again while the correct words were displayed on the screen, whether their response was correct or incorrect. The adaptive procedure used during the pretest was also employed in the training paradigm to measure the listener's time-compression threshold targeting 50% performance.

#### 2.3.3 Posttest session

After completing the five-session training program, each listener performed a posttest session either the same day or up to 3 days after the final training session. The posttest session consisted of the identical speech recognition tests and procedures that were followed in the pretest session.

## 3. Results

**Fig. 1.**
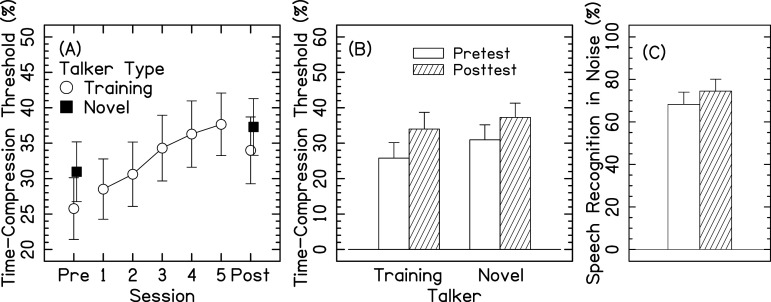
Average time-compression thresholds for 14 CI listeners. Error bars represent ±1 standard error. (A) shows the time-compression thresholds for the pretest, posttest, and each training session. Performance for the stimuli for the talker that was used in the training is shown by the open circles. Performances for the stimuli for the novel talker in pretest and posttest are depicted by the closed squares. For clarity, (B) shows the time-compression thresholds for just the pretest and posttest. (C) shows the pretest and posttest speech recognition scores for AzBio sentences presented in +10-dB SNR noise.

The benefit of training, as assessed by comparing pretest and posttest performance on the IEEE sentences, is shown in Fig. 1. Average time-compression thresholds increased, indicating better performance, across the five training sessions [Fig. 1(A)]. Individual data are available as supplementary material.

The time-compression threshold data were analyzed statistically with a linear mixed effects model (*R* version 4.3.1) using the buildmer (version 2.10.1) and lme4 (version 1.1-34) packages (R Development Core Team, 2023). The original model included: categorical variables of test time [pretest (reference), posttest] and talker type [trained (reference), novel], continuous variables of age and duration of deafness (both *z* transformed), and random intercepts by listener. The number of CIs worn by the listeners was omitted from the final reported model because there were no significant differences in all speech recognition scores across the two groups (*p* > 0.05 for all seven comparisons; two-sample two-tail *t*-tests assuming equal variances, uncorrected for multiple comparisons), and there were no significant effects or interactions when number of CIs was included. The final best fitting model describing how the time-compression thresholds changed between pretest and posttest, displayed in Fig. 1(B), is reported in Table 2. Time-compression thresholds increased by 8.2% (*p* < 0.0001) between pretest and posttest. The time-compression thresholds were 5.2% higher for the novel talker compared to the training talker (*p* = 0.004). The test time × talker interaction was not significant (*p* = 0.428). Age and duration of deafness were not significant (*p* > 0.05 for both) and not retained in the final model selection. In addition, age was not significantly correlated with pretest or posttest time-compression thresholds for the training talker, novel talker, or average of the two talkers (Pearson-product correlations, *p* > 0.05 for all six comparisons, uncorrected for multiple comparisons).

**Table 2.** Results of the final linear mixed effects model.

Fixed effects	Estimate	Standard error	Degrees of freedom (df)	*t*	*p*
Intercept	25.8	4.33	14.575	5.95	<0.0001
Test time	8.2	1.68	39	4.91	<0.0001
Talker	5.2	1.68	39	3.10	0.004
Test time × talker	−1.9	2.37	39	−0.80	0.428
Random effects	Variance				
Subject	243.1				

Figure 1(C) shows generalization of training with time-compressed sentences on the change in speech recognition for AzBio sentences presented in noise at +10 dB SNR. Speech recognition in noise increased by 6.3 ± 2.1% (paired two-sample two-tailed *t*-test, *p* = 0.005). A separate multiple regression was performed to determine if the change in speech recognition in noise (posttest − pretest) was predicted by age, duration of deafness, CNC word score, and change in time-compression threshold (posttest − pretest). None of these factors were predictive of the change in speech recognition in noise (*p* > 0.05 for all).

## 4. Discussion

The purpose of the study was to evaluate the benefit of auditory training with time-compressed speech for adult CI listeners over multiple adaptive training sessions. We hypothesized that CI listeners would benefit from adaptive auditory training, where larger benefits are observed in younger listeners compared to older listeners. We also hypothesized that improvements in time-compressed speech recognition would generalize to improvements in speech recognition in noise.

A comparison of pretest to posttest performance demonstrated a significant 5% average improvement in time-compressed speech recognition thresholds [Figs. 1(A) and 1(B)]. A 5% improvement in time-compressed speech recognition threshold would move trained older and middle-aged CI listeners closer to the performance of untrained younger CI listeners (Tinnemore *et al.*, 2022). Furthermore, a significant 6.3% improvement in speech recognition in noise was observed between pretest and posttest [Fig. 1(C)]. This tentatively supports our hypothesis that the benefit of time-compressed speech training generalizes to improved recognition of speech in noise; however, the lack of a significant correlation between improvements in time-compressed speech recognition and speech recognition in noise will require further investigation. Transfer of learning to untrained stimuli in older listeners can be limited (Bieber and Gordon-Salant, 2021).

Contrary to our hypotheses, age and duration of deafness were not significant predictors of the improvements in time-compressed speech recognition and speech recognition in noise (these terms were not retained in the final model in Table 2 and the multiple regression). Therefore, the findings support the benefit of auditory training in adult CI listeners, which is consistent with several other reports (Fu and Galvin, 2008; Dornhoffer *et al.*, 2024). Other forms of training, such as through daily practice and CI use, also seem to improve outcomes with CIs across the lifespan (DeFreese *et al.*, 2023). It is unclear if adaptive training that targets temporal-processing abilities differs from other more traditional training approaches, including speech recognition in noise (Fu and Galvin, 2008). It remains the case, however, that training that targets temporal-processing abilities specifically for older CI users has the potential to be particularly beneficial because it targets a pervasive auditory limitation of older people. Moreover, large gains can be observed in older acoustic-hearing listeners with a training regimen focused on auditory temporal processing (Anderson *et al.*, 2022).

Similar to previous research, the present study tested listeners at multiple sessions and evaluated performance over time (Goldsworthy and Shannon, 2014; Bissmeyer *et al.*, 2020). For the current study, a control group was not included. Future work, including active and passive control groups, is important to determine if the improvements observed were associated with the specific adaptive time-compressed speech training task, with an auditory training task, or with procedural learning. Although the statistical power was sufficient to show improvements in speech recognition from pretest to posttest, the sample size likely limited the strength of the analysis of demographic factors such as age and duration of deafness. Furthermore, it is not clear that improvements in time-compression thresholds saturated after five sessions [Fig. 1(A)], suggesting the possibility that listeners could have benefited from additional training sessions. Finally, it would be beneficial to determine if the listeners maintained their benefits over time (e.g., Dornhoffer *et al.*, 2024) by adding a long-term retention measurement several months after the training was completed.

Further study of different auditory training approaches and parameters is warranted to maximize benefits for CI listeners, particularly if age-related temporal-processing deficits can be remediated with targeted training. In younger and older acoustic-hearing listeners, rapid adaptation occurs with presentation of time-compressed speech, even in a single listening session. Several studies show that the rate of rapid adaptation to time-compressed speech is comparable across the lifespan (Peelle and Wingfield, 2005; Golomb *et al.*, 2007), which is consistent with our results in CI listeners in the current auditory training study over multiple sessions. Some reports, however, show smaller learning effects for older compared to younger acoustic-hearing listeners in longer-term auditory training (Manheim *et al.*, 2018).

The results clearly showed that the listeners exhibited significant improvement in recognition of time-compressed speech not only for the talker used in training but also for a novel talker [Fig. 1(B)]. It is assumed that training with time-compressed speech engenders auditory learning of abbreviated acoustic speech cues (Golomb *et al.*, 2007), improves a listener's attention to the speech materials, and/or improves general auditory temporal-processing abilities, as demonstrated with nonspeech stimuli in acoustic-hearing listeners (Anderson *et al.*, 2022) and CI listeners (Goldsworthy and Shannon, 2014; Bissmeyer *et al.*, 2020). However, without a control group, it is not possible to determine if primarily perceptual or procedural learning occurred.

In conclusion, this preliminary report indicates that targeted auditory training for CI listeners enhances perception of rapid acoustic stimuli, especially with time-compressed speech. Older CI listeners appear to benefit from this form of training as much as younger CI listeners. Building on the current findings, future studies should clarify the mechanism, generalization, and long-term retention for time-compressed speech training in younger and older CI users. Such endeavors may be promising for aural rehabilitation and improving understanding of challenging speech signals by adult CI listeners.

## Supplementary Material

See the supplementary material for individual data.

## Data Availability

The data that support the findings of this study are available from the corresponding author upon reasonable request.

## References

[1] Anderson, S. , DeVries, L. , Smith, E. , Goupell, M. J. , and Gordon-Salant, S. (2022). “ Rate discrimination training may partially restore temporal processing abilities from age-related deficits,” J. Assoc. Res. Otolaryngol. 23, 771–786. 10.1007/s10162-022-00859-x 35948694 PMC9365219

[2] Anderson, S. , Parbery-Clark, A. , White-Schwoch, T. , and Kraus, N. (2012). “ Aging affects neural precision of speech encoding,” J. Neurosci. 32, 14156–14164. 10.1523/JNEUROSCI.2176-12.2012 23055485 PMC3488287

[3] ANSI (2018). *American National Standard for Specification for Audiometers* ( American National Standards Institute, New York).

[4] Bieber, R. E. , and Gordon-Salant, S. (2021). “ Improving older adults' understanding of challenging speech: Auditory training, rapid adaptation and perceptual learning,” Hear. Res. 402, 108054. 10.1016/j.heares.2020.108054 32826108 PMC7880302

[5] Bissmeyer, S. R. S. , Hossain, S. , and Goldsworthy, R. L. (2020). “ Perceptual learning of pitch provided by cochlear implant stimulation rate,” PLoS One 15, e0242842. 10.1371/journal.pone.0242842 33270735 PMC7714175

[6] Blamey, P. J. , Artieres, F. , Başkent, D. , Bergeron, F. , Beynon, A. , Burke, E. , Dillier, N. , Dowell, R. , Fraysse, B. , Gallego, S. , Govaerts, P. J. , Green, K. , Huber, A. M. , Kleine-Punte, A. , Maat, B. , Marx, M. , Mawman, D. , Mosnier, I. , O'Connor, A. F. , O'Leary, S. , Rousset, A. , Schauwers, K. , Skarzynski, H. , Skarzynski, P. H. , Sterkers, O. , Terranti, A. , Truy, E. , Van de Heyning, P. , Venail, F. , Vincent, C. , and Lazard, D. S. (2013). “ Factors affecting auditory performance of postlinguistically deaf adults using cochlear implants: An update with 2251 patients,” Audiol. Neurotol. 18, 36–47. 10.1159/000343189 23095305

[7] Boersma, P. , and Weenink, D. (2013). “ Praat: Doing phonetics by computer (version 6.3.17) [computer program],” available at http://www.praat.org/ (Last viewed 10 September 2023).

[8] Canfarotta, M. W. , O'Connell, B. P. , Buss, E. , Pillsbury, H. C. , Brown, K. D. , and Dillon, M. T. (2020). “ Influence of age at cochlear implantation and frequency-to-place mismatch on early speech recognition in adults,” Otolaryngol. Head Neck Surg. 162, 926–932. 10.1177/0194599820911707 32178574 PMC8590812

[9] DeFreese, A. J. , Lindquist, N. R. , Shi, L. , Holder, J. T. , Berg, K. A. , Haynes, D. S. , and Gifford, R. H. (2023). “ The impact of daily processor use on adult cochlear implant outcomes: Reexamining the roles of duration of deafness and age at implantation,” Otol. Neurotol. 44, 672–678. 10.1097/MAO.0000000000003920 37367733 PMC10524754

[10] Dornhoffer, J. R. , Shannon, C. , Schvartz-Leyzac, K. C. , Dubno, J. R. , and McRackan, T. R. (2024). “ Computer-based auditory training by new adult cochlear implant recipients is associated with durable improvements in cochlear implant quality of life,” Ear Hear. (published online). 10.1097/AUD.0000000000001486 PMC11178477 38351509

[11] Frisina, D. R. , and Frisina, R. D. (1997). “ Speech recognition in noise and presbycusis: Relations to possible neural mechanisms,” Hear. Res. 106, 95–104. 10.1016/S0378-5955(97)00006-3 9112109

[12] Fu, Q. J. , and Galvin, J. J., III (2008). “ Maximizing cochlear implant patients' performance with advanced speech training procedures,” Hear. Res. 242, 198–208. 10.1016/j.heares.2007.11.010 18295992 PMC2603139

[13] Füllgrabe, C. , Moore, B. C. , and Stone, M. A. (2015). “ Age-group differences in speech identification despite matched audiometrically normal hearing: Contributions from auditory temporal processing and cognition,” Front. Aging Neurosci. 6, 347. 10.3389/fnagi.2014.00347 25628563 PMC4292733

[14] Goldsworthy, R. L. , and Shannon, R. V. (2014). “ Training improves cochlear implant rate discrimination on a psychophysical task,” J. Acoust. Soc. Am. 135, 334–341. 10.1121/1.4835735 24437773 PMC3985914

[15] Golomb, J. D. , Peelle, J. E. , and Wingfield, A. (2007). “ Effects of stimulus variability and adult aging on adaptation to time-compressed speech,” J. Acoust. Soc. Am. 121, 1701–1708. 10.1121/1.2436635 17407906

[16] Gordon-Salant, S. , and Fitzgibbons, P. J. (2001). “ Sources of age-related recognition difficulty for time-compressed speech,” J. Speech. Lang. Hear. Res. 44, 709–719. 10.1044/1092-4388(2001/056) 11521766

[17] Ji, C. , Galvin, J. J., III , Xu, A. , and Fu, Q. J. (2013). “ Effect of speaking rate on recognition of synthetic and natural speech by normal-hearing and cochlear implant listeners,” Ear Hear. 34, 313–323. 10.1097/AUD.0b013e31826fe79e 23238527 PMC3610785

[18] Kirby, A. E. , and Middlebrooks, J. C. (2012). “ Unanesthetized auditory cortex exhibits multiple codes for gaps in cochlear implant pulse trains,” J. Assoc. Res. Otolaryngol. 13, 67–80. 10.1007/s10162-011-0293-0 21969022 PMC3254721

[19] Kujawa, S. G. , and Liberman, M. C. (2015). “ Synaptopathy in the noise-exposed and aging cochlea: Primary neural degeneration in acquired sensorineural hearing loss,” Hear. Res. 330, 191–199. 10.1016/j.heares.2015.02.009 25769437 PMC4567542

[20] Lenarz, M. , Sonmez, H. , Joseph, G. , Buchner, A. , and Lenarz, T. (2012). “ Cochlear implant performance in geriatric patients,” Laryngoscope 122, 1361–1365. 10.1002/lary.23232 22539093

[21] Manheim, M. , Lavie, L. , and Banai, K. (2018). “ Age, hearing, and the perceptual learning of rapid speech,” Trends Hear. 22, 2331216518778651. 10.1177/2331216518778651 29877142 PMC5992806

[22] Middlebrooks, J. C. , and Snyder, R. L. (2010). “ Selective electrical stimulation of the auditory nerve activates a pathway specialized for high temporal acuity,” J. Neurosci. 30, 1937–1946. 10.1523/JNEUROSCI.4949-09.2010 20130202 PMC2828779

[23] Nasreddine, Z. S. , Phillips, N. A. , Bédirian, V. , Charbonneau, S. , Whitehead, V. , Collin, I. , Cummings, J. L. , and Chertkow, H. (2005). “ The Montreal Cognitive Assessment, MoCA: A brief screening tool for mild cognitive impairment,” J. Am. Geriatr. Soc. 53, 695–699. 10.1111/j.1532-5415.2005.53221.x 15817019

[24] Peelle, J. E. , and Wingfield, A. (2005). “ Dissociations in perceptual learning revealed by adult age differences in adaptation to time-compressed speech,” J. Exp. Psychol. Hum. Percept. Perform. 31, 1315–1330. 10.1037/0096-1523.31.6.1315 16366792

[25] R Development Core Team (2023). “ R: A language and environment for statistical computing (version 4.3.1),” available at https://www.R-project.org/ (Last viewed 10 October 2023).

[26] Rothauser, E. H. , Chapman, W. D. , Guttman, N. , Silbiger, H. R. , Hecker, M. H. L. , Urbanek, G. E. , Nordby, K. S. , and Weinstock, M. (1969). “ IEEE recommended practice for speech quality measurements,” IEEE Trans. Acoust. Speech Signal Process. 17, 225–246. 10.1109/TAU.1969.1162058

[27] Schafer, E. C. , Pogue, J. , and Milrany, T. (2012). “ List equivalency of the AzBio sentence test in noise for listeners with normal-hearing sensitivity or cochlear implants,” J. Am. Acad. Audiol. 23, 501–509. 10.3766/jaaa.23.7.2 22992257

[28] Spahr, A. J. , Dorman, M. F. , Litvak, L. M. , Van Wie, S. , Gifford, R. H. , Loizou, P. C. , Loiselle, L. M. , Oakes, T. , and Cook, S. (2012). “ Development and validation of the AzBio sentence lists,” Ear Hear. 33, 112–117. 10.1097/AUD.0b013e31822c2549 21829134 PMC4643855

[29] Tinnemore, A. R. , Montero, L. , Gordon-Salant, S. , and Goupell, M. J. (2022). “ The recognition of time-compressed speech as a function of age in listeners with cochlear implants or normal hearing,” Front. Aging Neurosci. 14, 887581. 10.3389/fnagi.2022.887581 36247992 PMC9557069

